# O008. Improvement in clinical governance of chronic headache by an information network: HealthSOAF - Calabria Headache Network pilot study

**DOI:** 10.1186/1129-2377-16-S1-A72

**Published:** 2015-09-28

**Authors:** Rosario Iannacchero, Maurizio Cipolla, Amerigo Costa, Domenico Conforti

**Affiliations:** Centre for Headache and Adaptive Disorders, Unit of Neurology, Department of Neuroscience and Sense Organs, Azienda Ospedaliera “Pugliese-Ciaccio”, Catanzaro, Italy; Unit of Continuing Primary Care of Catanzaro Lido, Azienda Sanitaria Provinciale, Catanzaro, Italy; Laboratory of Decision Engineering for Health Care Delivery, Department of Mechanical, Energy and Management Engineering, University of Calabria, Rende, Italy

## Background

**Figure 1:**
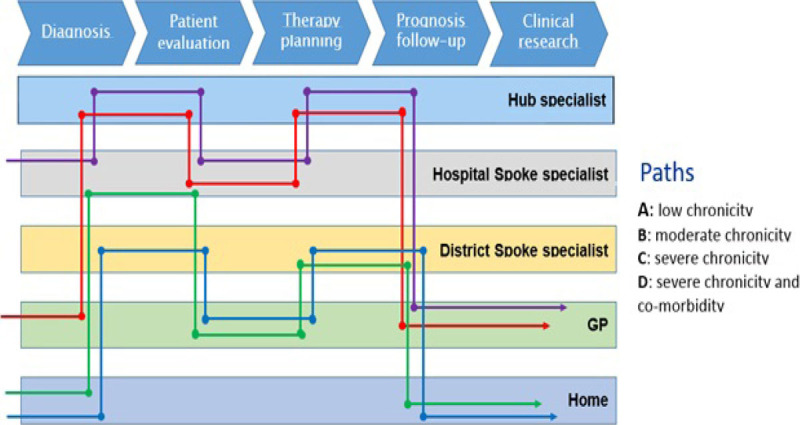
Diagnostic and therapeutic paths per clinical scenarios managed by HealthSOAF in the Calabria Headache Network.

Good clinical governance of headache implies efficient and accessible diagnostic and therapeutic paths involving health care at different levels[1]. Often clinicians do not appropriately assess and treat headache. Information and communication technologies might play a key role in improving access, quality, efficiency and prevention in health care. HealthSOAF (Service-Oriented Architecture Framework) is a networking and interoperability technological platform aimed to assist multiple level health care access and decision making. Its first real testing scenario in Europe has been the Headache Network in the Italian Region of Calabria targeting to assist clinicians at different levels of health care to correctly diagnose, manage and refer headache patients (Figure 1).

## Materials and methods

From November 2014 to March 2015, volunteer nodes in the Calabria Headache Network (10 primary care general practitioners in Catanzaro Lido, Borgia and Soverato; 3 secondary care neurologists in Catanzaro Lido - spokes; 1 multidisciplinary team tertiary care at the regional Centre for Headache and Adaptive Disorders at the Pugliese-Ciaccio Hospital in Catanzaro - hub) shared the HealthSOAF software client and network access. We retrieved epidemiological and referral data and compared them with preexisting information and estimation about the area.

## Results

One hundred and ninety-seven patients accessing the primary care units in the considered period obtained a diagnosis of headache. Nineteen (9.64%) diagnosed as secondary headache were referred to the Emergency Rooms. Seventy-four patients (37.56%) diagnosed as episodic primary headache were managed at the primary care level. Thirty-six patients (18.27%) were managed by the general practitioner and outpatient neurologist (episodic primary headache). Sixty-eight patients (34.52%) were referred to the Centre for Headache and Adaptive Disorders as chronic headache cases. Compared to preexisting data, this marked an improvement in access to headache care and reduction of inappropriate referrals to the Centre for Headache (pre: 15.42%; post: 7.35%).

## Conclusions

The use of the HealthSOAF platform in this experimental pilot is associated with enhanced diagnostic correctness and access to tailored headache services in the considered area, suggesting that network-based clinical decision support informational tools can improve the clinical governance of headache.

Written informed consent to publication was obtained from the patient(s).
